# Effect of ATRA and ATO on the expression of tissue factor in NB4 acute promyelocytic leukemia cells and regulatory function of the inflammatory cytokines TNF and IL-1β

**DOI:** 10.1007/s00277-017-2970-5

**Published:** 2017-03-25

**Authors:** Sylvie Dunoyer-Geindre, Anne-Sophie Rivier-Cordey, Olga Tsopra, Thomas Lecompte, Egbert K. O. Kruithof

**Affiliations:** 1 Division of Angiology and Hemostasis, Department of Medical Specialties, University Hospital of Geneva, Geneva, Switzerland; 2 Faculty of Medicine of the University of Geneva, Geneva, Switzerland; 3 Division of Hematology, Department of Medical Specialties, University Hospital of Geneva, Rue Gabrielle Perret-Gentil 4, 1211 Geneva, Switzerland

**Keywords:** Acute promyelocytic leukemia, Tissue factor, Tumor necrosis factor, Interleukin 1 beta, All-*trans* retinoic acid, Arsenic trioxide

## Abstract

The characteristic hemorrhages of acute promyelocytic leukemia (APL) are caused in part by the high expression of tissue factor (TF) on leukemic cells, which also produce TNF and IL-1β, proinflammatory cytokines known to increase TF in various cell types. Exposure of NB4 cells, an APL cell line, to all-*trans* retinoic acid (ATRA) or arsenic trioxide (ATO) rapidly and strongly reduced TF mRNA. Both drugs also reduced TNF mRNA, but later, and moreover increased IL-1β mRNA. The effect on procoagulant activity of cells and microparticles, as measured with calibrated automated thrombography, was delayed and only partial at 24 h. TNF and IL-1β inhibition reduced TF mRNA and activity only partially. Inhibition of the inflammatory signaling intermediate p38 reduced TF mRNA by one third but increased TNF and IL-1β mRNA. NF-κB inhibition reduced, within 1 h, TF and TNF mRNA but did not change IL-1β mRNA, and rapidly and markedly reduced cell survival, with procoagulant properties still being present. In conclusion, although we provide evidence that TNF, IL-1β, and their signaling intermediates have a regulatory function on TF expression by NB4 APL cells, the effect of ATRA and ATO on TF can only partially be accounted for by their impact on these cytokines.

**Electronic supplementary material:**

The online version of this article (doi:10.1007/s00277-017-2970-5) contains supplementary material, which is available to authorized users.

## Introduction

The persistent and worrisome hallmark of acute promyelocytic (M3) leukemia (APL) is the high risk of severe, often fatal, bleeding complications [1–6]. Pathogenesis of the coagulopathy is complex and includes an insufficient production of platelets, as well as disseminated intravascular coagulation (DIC) [2, 6–9], caused, at least in part, by tissue factor (TF) expressed on the leukemia cells and on leukemia cell-derived microparticles expressing TF and procoagulant phosphatidylserine on their surface [10–14]. Fibrinolysis, mediated by t-PA bound to annexin 2 on the leukemia cell, is another important factor contributing to hemorrhagic complications [15].

Treatment of APL patients with all-*trans* retinoic acid (ATRA) or arsenic trioxide (ATO) leads, over a period of 1 to 3 weeks, to normalization of plasma concentrations of D-dimers and thrombin–antithrombin complexes [7, 8, 16, 17] and of TF mRNA in patient-derived bone marrow cells [8, 16, 18]. Studies performed with cultured bone marrow cells from APL patients revealed that exposure to ATRA reduced cell-associated procoagulant activity [19]. Experiments using NB4 cells, an APL cell line that presents the characteristic 15;17 chromosomal translocation, showed that exposure to ATRA or ATO resulted in a reduction of TF mRNA and antigen [18, 20–22] as well as of TF activity [12]. However, as therapy by ATRA or ATO (mostly) leads to APL cell apoptosis and thus generation of microparticles [10, 23], it is possible that ATRA-mediated differentiation of APL cells leads to a transient increase in procoagulant activities, despite its downregulating effect on TF mRNA [13]. A further factor that has to be taken into account is the production by APL cells of proinflammatory cytokines, such as TNF and IL-1β [24, 25]. This may be of clinical relevance because these cytokines are capable, among other properties, of increasing TF production in monocytes and endothelial cells and, considering that NB4 cells express TNF receptor 1 [26], could also contribute to TF production by APL cells.

In the present study, we used NB4 cells to investigate in more detail the time course of the effects of ATRA and ATO on TF activity and on expression of the proinflammatory cytokines TNF and IL-1β. In addition, we investigated to what extent TF production by NB4 cells depends on TNF and IL-1β they also produce and whether it is affected by interfering with the inflammatory signaling intermediates p38, jun kinase, and NF-κB. We observed that exposure of NB4 cells to ATRA led within 1 h to a reduction of TF mRNA and to a reduction of TNF mRNA but only after 6 h. Exposure to ATO also induced a reduction of TF and TNF mRNA, which was detectable only after 3 and 6 h, respectively. Both ATRA and ATO increased IL-1β mRNA several fold. A partial reduction in TF antigen and TF activity was evident only after 24 h of ATRA or ATO treatment. Inhibition of TNF and, to a lesser extent, of IL-1β only partially reduced TF mRNA. Inhibition of p38 reduced TF mRNA but strongly increased TNF and IL-1β mRNA, while inhibition of JNK had no effect on TF and TNF mRNA but reduced IL-1β mRNA. Inhibition of NF-κB reduced TF and TNF mRNA in NB4 cells with more than 50% within 1 h but also reduced cell survival with a half-life of approximately 6 h.

## Materials and methods

### Reagents

ATRA and ATO were from Sigma-Aldrich. Adalimumab (Humira®), a TNF activity-blocking antibody, was from Abbott Laboratories, and Anakinra (Kineret®), an IL-1 receptor antagonist, was from Swedish Orphan Biovitrum. BAY11-7085, an inhibitor of NF-κB, was from Biomol. The p38 inhibitors SB203580, SB202190, and Birb796 were from Sigma-Aldrich, Biomol, and Axon Medchem, respectively. The jun kinase inhibitor SP600125 was from Tocris Biosciences.

### Cell lines used

NB4 APL and HL-60 acute myeloid leukemia cells were obtained from the German Collection of Microorganisms and Cell Cultures (http://www.dsmz.de). NB4 cells contain the 15;17 chromosomal translocation and are used as a standard model for investigations on the mechanisms of cellular responses of APL cells, whereas HL60 cells lack the 15;17 chromosomal translocation. NB4 and HL60 cells were cultured in RPMI 1640 medium (Gibco) + 10% fetal bovine serum (Pan Biotech) and 1% of penicillin/streptomycin. U937-PR9 cells, stably transfected with DNA encoding the PML-RARα fusion protein under control of the metallothionin promoter, as well as the corresponding control U-937MT cells [27], were a kind gift from Prof. P.G. Pelicci (Department of Experimental Oncology, European Institute of Oncology, 20141 Milan, Italy). The cells were cultured at a density of 10^6^ cells/ml in RPMI 1640 medium + 10% fetal bovine serum and 1% of penicillin/streptomycin. Cells were used for up to ten passages after having been received. They were isolated by centrifugation for 5 min at 300×*g* and 4 °C and cell-derived microparticles by centrifugation of the cell supernatant for 5 min at 1500×*g* to remove cell debris followed by a further centrifugation for 30 min at 20,000×*g*. Cells and microparticles were suspended in Dulbecco’s phosphate-buffered saline (Gibco, cat. no. 14190-094). Coagulation studies with the supernatant prepared as just above described will be referred to as “microparticle-associated coagulant activities.” Those studies were thus performed with both cell suspension (50,000/ml) and microparticles (suspended in an equivalent volume as the cells).

### Quantitative reverse transcriptase real time PCR (qPCR) assay for TF, TNF, and IL-1β mRNA

Total cellular RNA was isolated using the TRIzol reagent (Invitrogen) and reverse transcribed using the Improm-II reverse transcriptase system from Promega. Thereafter, qPCR was performed as described previously [28] using the ΔΔCT method and GAPDH as the control housekeeping gene. The following forward and reverse primer sequences, respectively, were used for mRNA quantification by qPCR:

- GAPDH: GGTGAAGGTCGGAGTCAAC and CCATGGGTGGAATCATATTG
- TF: CTACTGTTTCAGTGTTCAAGCAGT and CAGTGCAATATAGCATTTGCAGTAGC
- TNF: CCCAGGCAGTCAGATCATCTTC and AGCTGCCCCTCAGCTTGA
- IL-1β: AAACAGATGAAGTGCTCCTTCCAGG and TGGAGAACACCACTTGTTGCTCCA
- CD11c: AGAGCTGTGATAAGCCAGTTCC and AATTCCTCGAAAGTGAAGTGTGT

### Flow cytometry analysis

TF and CD11c, a marker of granulocytic differentiation, were analyzed with flow cytometry using a BD Biosciences Accuri C6 flow cytometer. APC-labeled antibodies to TF (CD142) and its isotype (IgG1kappa) matched APC-labeled control antibodies (IgG-APC) were from eBioscience (cat. nos. 17-1429 and 17-4714, respectively) and phycoerythrin-labeled antibodies to CD11c and its isotype (IgG1kappa) matched PE-labeled control antibodies (IgG-PE) were from BD Pharmingen (cat. nos. 555392 and 555749, respectively). The cells were first washed in phosphate-buffered saline (PBS) + 1% bovine serum albumin (PBS/BSA) and 0.01% sodium azide. Then, 200,000 cells were incubated for 30 min at room temperature with 0.6 μl IgG-APC + 20 μl IgG-PE or with 5 μl CD142-APC + 20 μl CD11c-PE in a total volume of 50 μl PBS/BSA. Thereafter, 500 μl of PBS/BSA was added and the cells centrifuged for 5 min at 200×*g* and 4 °C. The cells were washed once with 500 μl of PBS/BSA and resuspended in 200 μl PBS/BSA. The cell suspension was stored on ice in the absence of light. Five minutes before flow cytometry analysis, 5 μl of 1 mg/ml of 7-amino actinomycin (7-AAD; R&D systems) was added to exclude dead cells from analysis.

### Coagulation assays

TF activity was quantified with a factor FXa generation assay [29], and overall coagulant activity was assessed with a thrombin generation assay (calibrated automated thrombography) [30]. The contribution of TF to activities measured in those assays was explored by comparing activities in the presence and absence of 10 μg/ml of a murine monoclonal anti-TF antibody (Ref 4509, Sekisui Diagnostics). This anti-TF antibody concentration was shown to be sufficient to completely block factor FXa generation activity on LPS-treated human endothelial cells [31].

#### Factor Xa generation assay

In short, hundred microliters of a suspension of cells (50,000/ml) or microparticles (suspended in an equivalent volume as the cells) was incubated with 5 μl of human coagulation factor X (at 3.4 μM; HCX-0500, Haematologic Technologies Inc.) and 10 μl of factor VIIa (at 50 nM, Novoseven, Novo Nordisk). The samples were incubated for 15 min at 37 °C and further FXa generation stopped by addition of 50 μl of 15 mM EDTA. Thereafter, 50 μl of the FXa chromogenic substrate SFXA-11 (Hyphen Biomed) was added at 3.9 mM and the mixture incubated for a further 15 min at 37 °C. After addition of 100 μl of 1 M H_2_SO_4_, the absorbance was read at 405 nm and compared to that of a standard curve made using different concentrations of bovine factor FXa (Hyphen Biomed). Results are expressed as relative to the activity of unexposed NB4 cells.

#### Calibrated automated thrombography (CAT) assay

This was done according to the manufacturer’s instructions using reagents and procedures provided by Diagnostica Stago. Suspensions of cells (50,000/ml) or microparticles (suspended in an equivalent volume as the cells) were mixed with normal plasma and fluorescence development measured with a Fluoroskan Ascent (Thermo electron corporation) microplate fluorimeter and analyzed using Thrombinoscope™ software (Diagnostica Stago).

## Statistical analysis

Data are presented as means ± standard error of the mean (SEM). The significance of differences was assessed using the paired Student’s *t* test.

## Results

### Effect of ATRA and ATO on mRNA levels of TF, TNF, IL-1β, and CD11c in NB4 and HL-60 cells

The inflammatory cytokines TNF and IL-1β are known to be produced by APL cells and are capable of increasing TF expression in some cell types. We first investigated in NB4 cells to what extent ATRA and ATO, which are nowadays standard therapeutics in APL patients, modified mRNA levels of TF, TNF, and IL-1β. In preliminary concentration response experiments we determined after 24-h incubation, the concentrations at which ATRA and ATO attained their maximal effect.

**Fig. 1:**
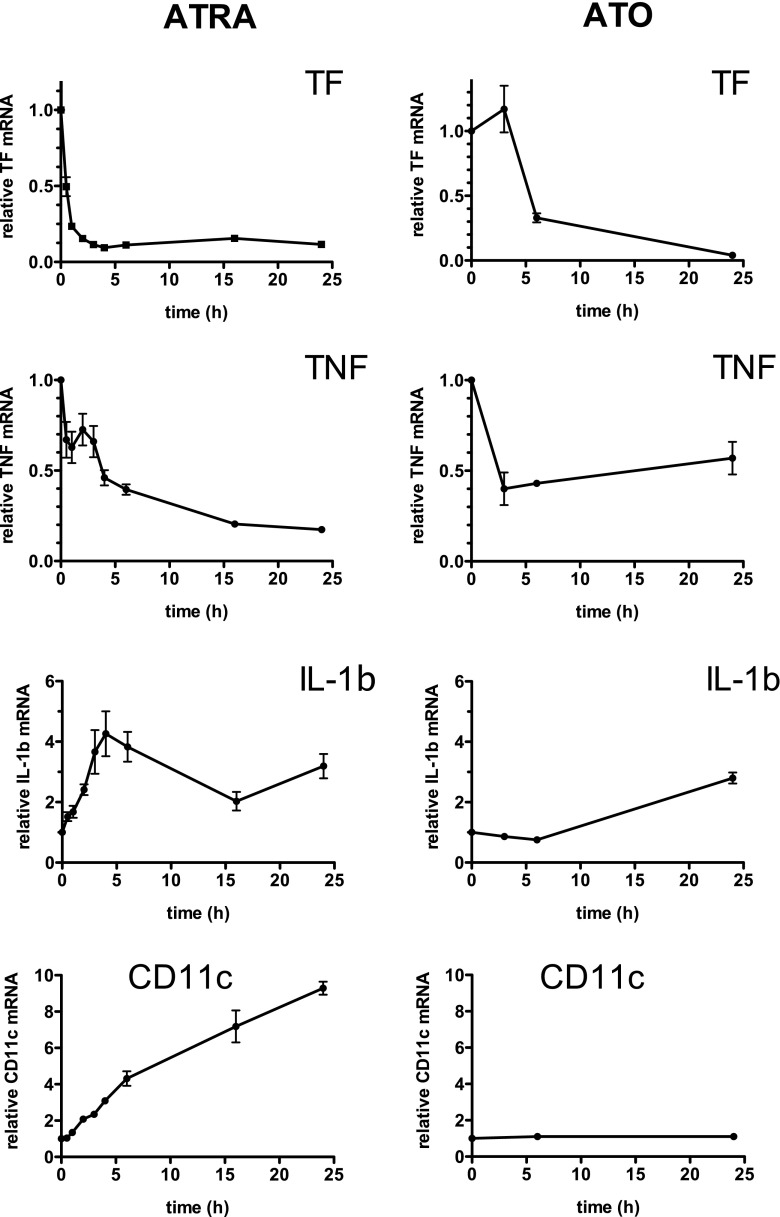
Time course of the effect of ATRA or ATO on mRNA levels of TF, TNF, IL-1β, and CD11c in NB4 cells. NB4 acute promyelocytic leukemia cells were exposed for the indicated time periods to 0.5 μM ATRA (*left*) or 5 μM ATO (*right*). Levels of mRNA were quantified by qPCR and expressed relative to that of the corresponding mRNA in control (untreated cells, incubated 24 h in medium alone) NB4 cells. The results are the means ± SEM of at least four independent experiments

With ATRA, in NB4 cells, we observed a maximal effect after 24 h at 0.5 μM. At this concentration, TF mRNA was reduced by 92 ± 2% (mean ± SEM, *n* = 4) and TNF mRNA by 88 ± 1%. A time–response curve performed with 0.5 μM ATRA showed that ATRA attained its maximal effect on TF mRNA already within 1 h, whereas its inhibitory effect on TNF mRNA was detectable only after 4 h and attained its maximal effect of more than 80% inhibition only after 15 h (Fig. 1, left). In contrast with its inhibitory effect on TF and TNF mRNA, 0.5 μM ATRA increased IL-1β mRNA by 4.3 ± 0.7-fold, already within 4 h.

To assess whether these results were specific to NB4 cells, the effects of ATRA on the expression of TF, TNF, and IL-1β were also investigated in HL-60 cells, another myelocytic leukemic cell line widely used for such in vitro studies. After 3-h ATRA treatment, TF mRNA was reduced by at most by 35%, whereas no changes in TNF mRNA were observed (not shown). Levels of IL-1β mRNA in HL-60 cells were below the detection limit.

With ATO and NB4 cells, we observed a maximal effect at 5 μM. At this concentration, both TF and TNF mRNA were reduced by 60% after 6 h incubation. No further reduction was observed at 24 h for TNF mRNA, whereas TF mRNA was almost undetectable after 24 h (Fig. 1, right). ATO at 5 μM increased IL-1β mRNA levels up to threefold but only after 24 h. In HL-60 cells, ATO increased TF mRNA by up to twofold (not shown) and had no effect on TNF mRNA.

To determine whether the effects of exposure of NB4 cells to ATRA and ATO were correlated with their differentiation, we performed mRNA analysis and flow cytometry analysis for CD11c, a marker for promyelocytic cell differentiation. Exposure to ATRA resulted in a gradual increase in CD11c mRNA (fourfold at 6 h and tenfold at 24 h) (Fig. 1, left). CD11c antigen at the cell surface, as assessed by flow cytometry, was only detectable after 16 and 24 h of ATRA treatment (not shown). The effect of ATRA on TF precedes its effect on cell maturation, as the maximal effect of ATRA on TF mRNA was obtained within 1 h. In contrast to ATRA, and as expected, ATO had no effect on CD11c mRNA (Fig. 1, right) and CD11c antigen (not shown).

### Effect of the PML-RARα fusion protein on expression of TF, TNF, and IL-1β

**Fig. 2:**
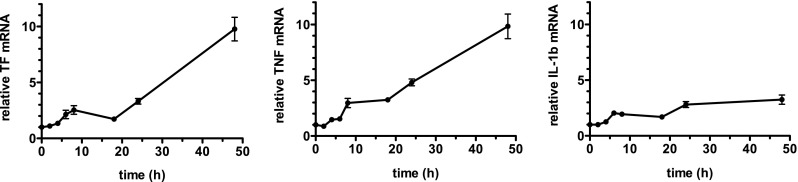
Effect of the PML-RARα fusion protein on mRNA levels of TF, TNF, and IL-β in U-937-PR9 cells. U937-PR9 cells, in which PML-RARα production is under control of the metallothionin promoter, were incubated for up to 48 h with 100 μM of Zn^2+^ to stimulate the production of the PML-RARα fusion protein. Levels of mRNA for TF (*left*), TNF (*center*), and IL-1β (*right*) were quantified by qPCR and expressed relative to that of the corresponding mRNA in control (untreated cells, incubated 24 h in medium alone) U937-PR9 cells. The results are the means ± SEM of at least four independent experiments

As ATRA and ATO are both capable of reducing the PML-RARα fusion protein [32], we investigated the effect of specifically increasing PML-RARα on mRNA levels of TF, TNF, and IL-1β. For this, we used the U937-PR9 cell line in which PML-RARα production is under control of the metallothionin promoter. In the presence of 100 μM of Zn^2+^, these cells produce PML-RARα, whereas this fusion protein is not made in the absence of Zn^2+^ [27]. This allows thus the direct comparison of the effect of PML-RARα on expression of TF, TNF, and IL-1β. Zn^2+^ treatment of U937-PR9 cells resulted in an almost tenfold increase of both TF and TNF mRNA after 48 h (Fig. 2, left and center). At this time point, a threefold increase in IL-1β mRNA was observed (Fig. 2, right). No changes in TF and TNF mRNA were observed after Zn^2+^ treatment of the control U937-MT cell line, which is stably transfected with an empty plasmid containing a metallothionin promoter (data not shown). We then studied whether ATO, which inactivates the PML-RARα fusion protein, had an effect on TF mRNA in U937-PR9 cells pretreated for 48 h with 100 μM Zn^++^. We observed that incubation of these cells with 1 μM or 5 μM ATO reduced TF mRNA by 59 ± 7% and 89 ± 2% for 5 μM ATO, respectively (mean ± SE of three independent experiments).

### Effect of ATRA and ATO on TF antigen and activity of NB4 cells and of their microparticles

**Fig. 3:**
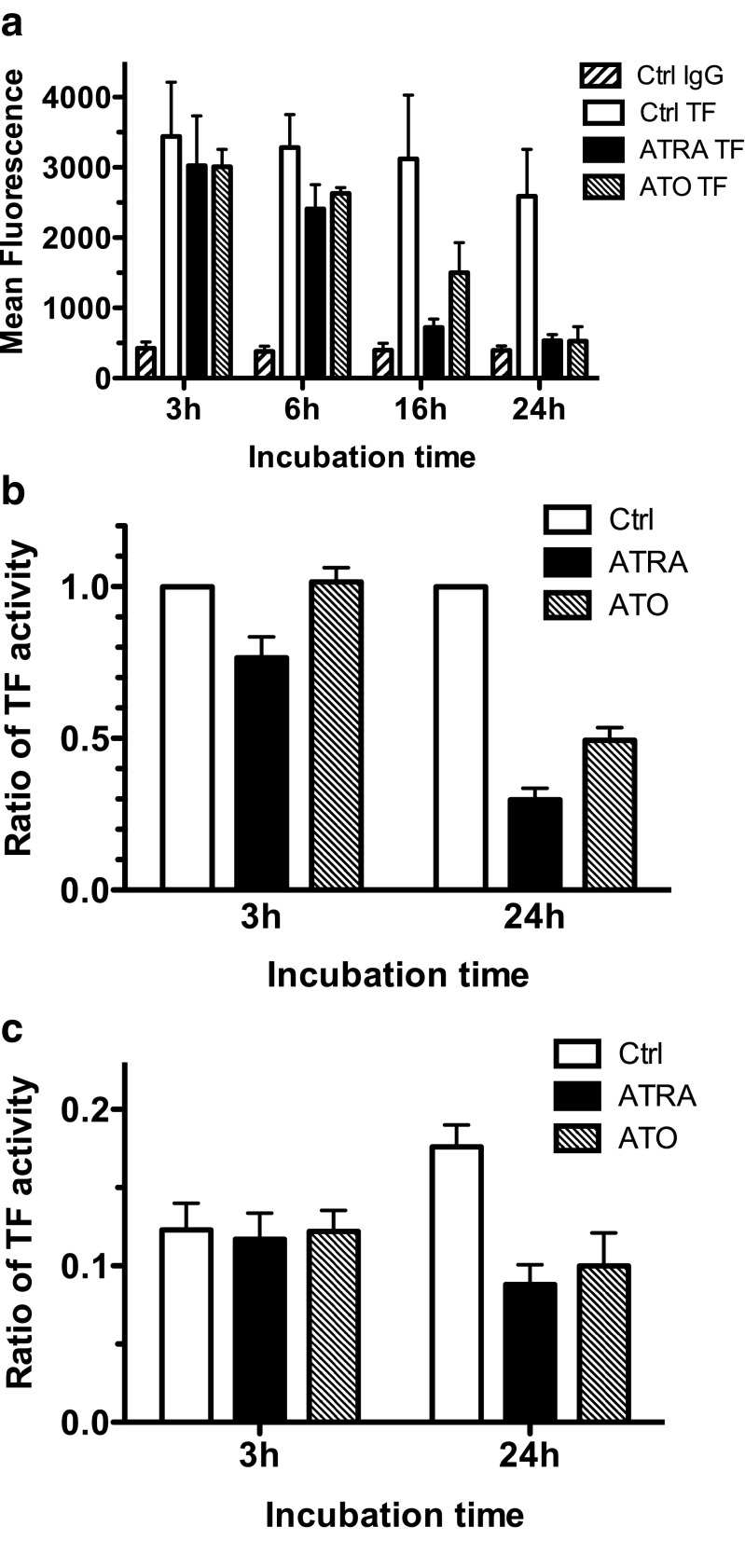
Effect of ATRA and ATO on TF antigen and activity of NB4 cells and NB4-cell-derived microparticles. **a** NB4 cells were exposed for the indicated time periods to 0.5 μM ATRA or 5 μM ATO. After the indicated time periods, TF antigen on the NB4 cell surface was analyzed by flow cytometry. “Ctrl IgG” are flow cytometry results obtained with an APC-labeled isotope matched control antibody and “Ctrl TF” are flow cytometry results obtained with APC-labeled antibodies on unexposed NB4 cells. Results are expressed as means ± SEM of at least four independent experiments. The reduction in TF antigen at 24 h in the ATRA or ATO treated cells with respect to the unexposed cells (Ctrl TF) was significant with *p* values below 0.01. **b** and **c** TF activity of NB4 cells (**b**) or NB4 cell-derived microparticles (**c**) after 3 or 24 h exposure to 0.5 μM ATRA or 5 μM ATO. All the results of **b** and **c** are expressed as ratio of TF activity as compared to that of unexposed NB4 cells at the same time point. The results are the means ± SEM of five to seven independent experiments. The reduction in TF activity at 24 h in the ATRA- or ATO-treated cells or derived microparticles with respect to the unexposed cells (Ctrl) or their microparticles was significant with *p* values below 0.01

Flow cytometry analysis was performed to assess TF antigen at the NB4 cell surface. We observed that both ATRA and ATO reduced TF antigen. A 26% and 19% decrease in TF antigen was observed after a 6-h exposure to ATRA or ATO, respectively. After 24 h of exposure to ATRA or ATO, hardly any TF antigen was seen at the NB4 cell surface. Indeed, the flow cytometry signal for TF was only marginally higher than the signal obtained with isotype-matched control antibodies (Fig. 3a).

We then analyzed the effects of ATRA and ATO on procoagulant activities expressed on NB4 cells and on NB4 cell-derived microparticles. We first determined the contribution of TF to NB4 cell or microparticle-associated FXa generation activity. We observed that co-incubation with 10 μg/ml of blocking anti-TF monoclonal antibody reduced NB4 cell- and microparticle-associated activity by 79% and 100%, respectively. A 3-h exposure to 0.5 μM ATRA reduced cell-associated FXa generation activity by 22%, whereas ATO had no effect (Fig. 3b). No differences in microparticle-associated activities were observed at this time point (Fig. 3c). After 24-h exposure, NB4 cell-associated activity was reduced by 69 ± 19% (*p* < 0.001) by ATRA and by 52 ± 12% (*p* < 0.002) by ATO (Fig. 3b). At this time point, ATRA and ATO reduced microparticle-associated TF activity by 50 ± 29% (*p* < 0.001) and 47 ± 28% (*p* < 0.002), respectively (Fig. 3c).

**Fig. 4:**
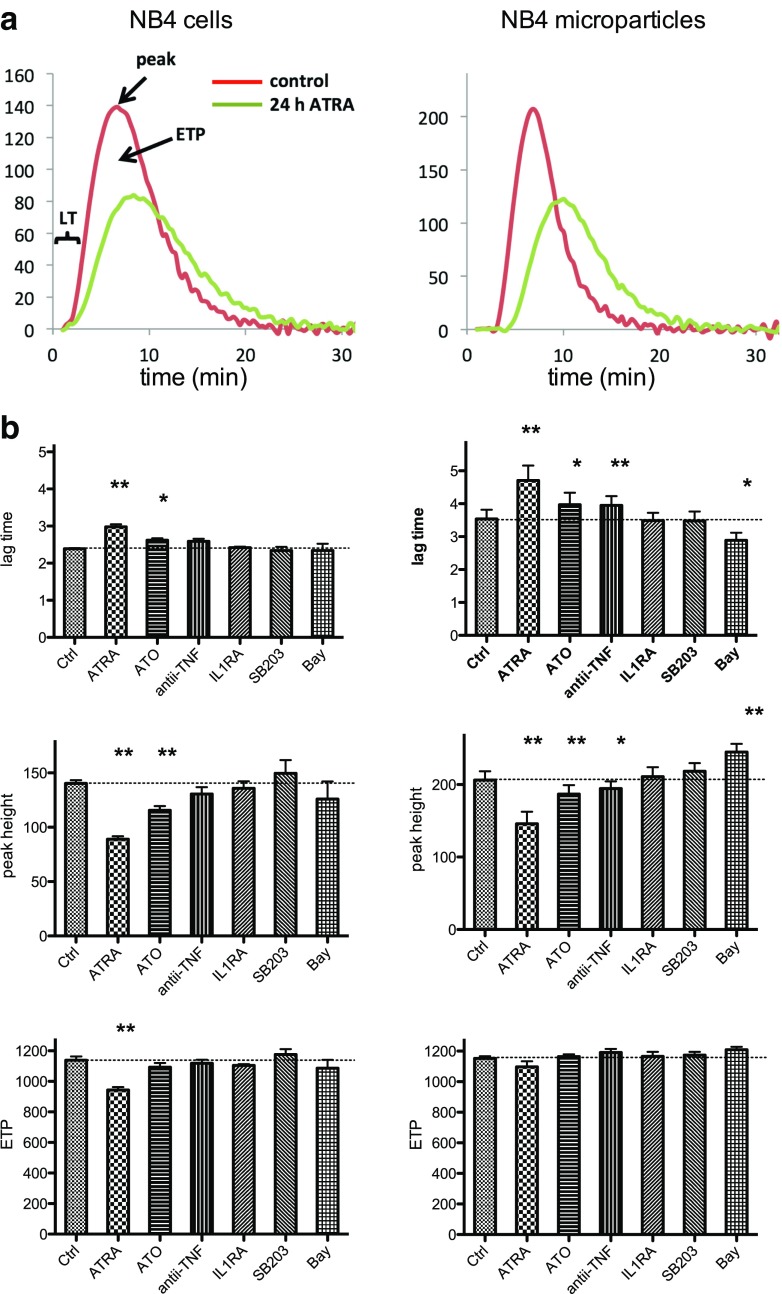
Effect of the agents investigated on procoagulant activities of NB4 cells or NB4 cell-derived microparticles as assessed with calibrated automated thrombography. NB4 cells were exposed for 24 h to the indicated agent and procoagulant activities of NB4 cells (*left*) or NB4 cell-derived microparticles (*right*) assessed with the CAT assay. **a** Typical thrombogram (CAT) of unexposed (*red*) or ATRA treated (*green*) NB4 cells or NB4 cell-derived microparticles. *LT* lag time (= time elapsed before thrombin burst), peak height (= maximal thrombin concentration), and ETP: area under the curve (= total amount of thrombin action). **b** Histograms of results obtained regarding lag time, peak height, and ETP with cells (*left*) or cell-derived microparticles (*right*) obtained from NB4 cells exposed to 0.5 μM ATRA, 5 μM ATO, 10 μg/ml of adalimumab (anti-TNF: a TNF inhibitory antibody), 10 μg/ml of anakinra (IL1RA: an IL-1 receptor antagonist), 10 μM SB203580 (SB203: a p38 inhibitor), or 100 μM BAY11-7085 (Bay: a NF-κB inhibitor). Results are presented as the means ± SEM of four to six independent experiments. Differences were analyzed using the paired Student’s *t* test. (*p < 0.05 and **p < 0.01)

We also analyzed with the CAT assay the effect of ATRA and ATO on procoagulant activities of NB4 cells and NB4 cell-derived microparticles. This assay takes into account the contribution of TF, anionic phospholipids, and any other potential players, even inhibitors such as thrombomodulin, expressed by NB4 cells [20, 33, 34], to thrombin generation [35], and is a more global and relevant assay than the FXa generation assay. With a 24-h incubation with ATRA, we observed an increase in the lag time (LT = time elapsed before the thrombin burst) and a decrease in the slope of the ascending part of the curve (= a reduction in the presence of anionic phospholipids), the peak height (peak = maximal thrombin concentration) and of the area under the curve (referred to as “endogenous thrombin potential”—ETP = total amount of thrombin action); this was observed both for cells and microparticles (Fig. 4a, b). Exposure to ATO also increased the lag time but to a lesser extent than ATRA (Fig. 4b) and also reduced peak height, but its effect on the area under the curve was minimal.

### Effect of inhibition of TNF or IL-1β on TF mRNA and procoagulant activity of NB4 cells

**Fig. 5:**
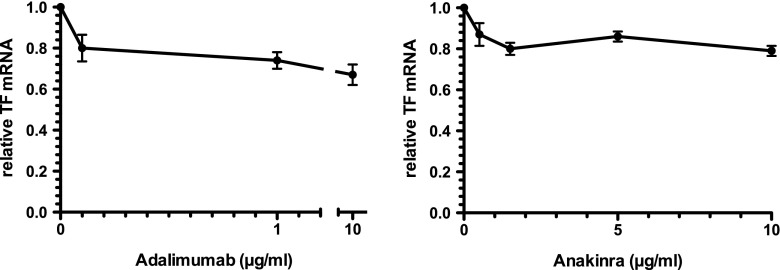
Effect of inhibition of TNF or IL-1β on TF mRNA levels in NB4 cells. NB4 cells were exposed for 24 h to various concentrations of adalimumab (a TNF inhibitory antibody) or anakinra (an IL-1 receptor antagonist) and TF mRNA levels were quantified by qPCR and expressed relative to that of the corresponding mRNA in control (unexposed cells, incubated in medium alone) NB4 cells. Results are expressed as means ± SEM of six independent experiments

The reduction in TF and TNF mRNA in ATRA- or ATO-treated NB4 cells, as well as the known ability of TNF to increase TF in monocytes and endothelial cells, raised the question as to a possible contribution of TNF to TF expression by these cells. Incubation of NB4 cells for 24 h with adalimumab, an inhibitory monoclonal antibody to TNF, resulted in a concentration-dependent reduction in TF mRNA with a maximum of 28% at 10 μg/ml (Fig. 5). Increasing adalimumab concentrations to 100 μg/ml did not lead to a further reduction in TF mRNA (data not shown).

As NB4 cells also produce IL-1β, we investigated, in addition, the effect of inhibiting this inflammatory cytokine. Incubation of NB4 cells with the IL-1β receptor antagonist anakinra resulted in a reduction of 20% at most (Fig. 5).

We also measured, using the CAT assay, the effect of TNF or IL-1β inhibition on procoagulant activities of NB4 cells and NB4 cell-derived microparticles. With anti-TNF, we only observed a small significant increase in lag time and a decrease in peak height for NB4 microparticles but no effect on area under the curve (Fig. 4b). Treatment with anakinra had no significant effect on any of the parameters of the CAT assay (Fig. 4b).

### Effect of inflammatory signaling intermediates on expression of TF, TNF, and IL-1β by NB4 cells

**Fig. 6:**
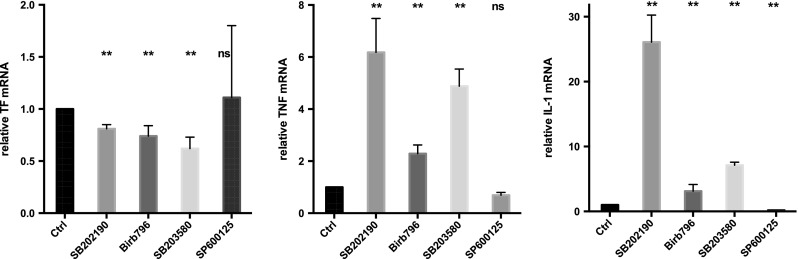
Effect of inhibition of p38 or jun kinase on mRNA levels of TF, TNF, or IL-1β in NB4 cells. NB4 cells were exposed for 24 h to 10 μM of the p38 inhibitors SB202190, Birb796, or SB203580 or 10 μM of the jun kinase inhibitor SP600126 and mRNA levels of TF, TNF, or IL-1β quantified by qPCR and expressed relative to that of the corresponding mRNA in control (unexposed cells, incubated in medium alone) NB4 cells. Results are expressed as means ± SEM of six independent experiments. Results obtained with the p38 inhibitors are significant with a *p* value <0.01

TNF and IL-1β exert their proinflammatory effects by signaling intermediates including p38 MAP kinase, jun kinase (JNK), and NF-κB. Inhibition of p38 with 10 μM of SB202190, Birb796, or SB203580 reduced TF mRNA in NB4 cells by 19 ± 4%, 26 ± 10%, and 38 ± 11%, respectively (Fig. 6). In contrast, these p38 inhibitors increased mRNA of TNF and IL-1β (Fig. 6). The strongest effect was seen with SB202190, which increased TNF mRNA and IL-1β mRNA by 6.2 ± 1.3-fold and 26.1 ± 4.1-fold, respectively. An intermediate effect was seen with SB203580 with a 4.9 ± 0.7 and 7.1 ± 0.5-fold increase in TNF and IL-1β mRNA, respectively, and the smallest effect with Birb796 with a 2.2 ± 0.3 and 3.1 ± 1.0 increase in TNF and IL-1β mRNA, respectively. Inhibition of JNK, using 10 μM of SP600125, had no effect on TF mRNA and reduced TNF and IL-1β mRNA by 31 ± 11% and 80 ± 2%, respectively (Fig. 6). We also measured, using the CAT assay, the effect of the p38 inhibitor SB203580 on procoagulant activities of NB4 cells and NB4 cell-derived microparticles. No significant changes in lag time, peak height, and area under the curve were observed (Fig. 4b).

Inhibition of NF-κB, using 100 μM of BAY11-7085, led to a very rapid reduction in TF and TNF mRNA. Minimal levels were observed already after 1 h (49% and 42%, respectively; Supplementary Fig. 1). BAY11-7085 had no effect on IL-1β mRNA. An unexpected finding was that BAY11-7085 had a strong effect on cell survival. After 6 h incubation with 100 μM of BAY11-7085, NB4 cell survival was only 42%, as compared to over 95% for untreated NB4 cells and after 24 h cell survival was less than 10%. In view of this, mRNA measurements are shown only for the time period up to 2 h. Incubation of NB4 cells for 24 h with BAY11-7085 had no effect on procoagulant activities of NB4 cells, as measured with the CAT assay, but reduced the lag time of and increased peak height NB4 cell-derived microparticles (Fig. 4b).

## Discussion

Human APL cells produce both TF and the inflammatory cytokines TNF and IL-1β. This study was undertaken to investigate the effect of ATRA and ATO on the expression of these proteins and to better understand the contribution of the inflammatory cytokines TNF and IL-1β, as well as the signaling intermediates p38, JNK, and NF-κB to TF expression by APL cells. For this, we used the NB4 human APL cell line, which has the 15;17 chromosomal translocation leading to the production of the PML-RARα fusion protein [36], that is characteristic for the majority of acute promyelocytic leukemia cases [37]. NB4 cells were chosen for this study, because the behavior of these cells was shown, in many studies, to be comparable to that of patient-derived primary APL cells [12, 18, 34, 38–44]. We also analyzed the effect of ATRA and ATO in HL-60 acute myeloid leukemia cells which lacks the 15;17 translocation [45]. Our results in NB4 cells show that ATRA and ATO reduce TF and TNF mRNA but increase IL-1β mRNA; that at the NB4 cell concentrations used (10^6^ cells/ml), TNF and to a lesser extent IL-1β partially contribute to TF expression; and that NF-κB and p38 contribute to TF expression. Our results that ATRA reduces TF mRNA and activity in NB4 cells are in agreement with previous results using this cell line [18, 20–22] and with results obtained with bone marrow cells from APL patients at different times after initiation of ATRA treatment [7, 19, 27]. ATRA and ATO both have similar effects on TF mRNA. However, ATRA reduces by 90% already within 1 h, whereas the effect of ATO on TF mRNA is evident only after 6 h.

In HL-60 cells, which lack the 15;17 chromosomal translocation, ATRA acted more slowly on TF mRNA and a maximal of 35% inhibition was obtained after 3 h, whereas ATO increased TF mRNA up to twofold. This implies that the effect of ATRA and ATO on TF expression is different in NB4 and HL-60 cells.

In NB4 cells, both ATRA and ATO induce the degradation of the PML-RARα fusion protein [32], which is capable of stimulating an increased TF expression [18]. Our finding that Zn^2+^ stimulation of U937-PR9 cells strongly increases TF and TNF mRNA (Fig. 2) and that ATO treatment of these Zn^2+^ stimulated cells reduced TF mRNA is compatible with the hypothesis that ATRA and ATO reduced TF and TNF via the degradation of PML-RARα. However, the increase in IL-1β also induced by Zn^2+^ stimulation of U937-PR9 cells (Fig. 2) contrasts with the fact that neither ATRA nor ATO reduced this cytokine. In addition, the rapidity of the effect of ATRA on TF expression rather suggests a primary action that is independent of PML-RARα degradation. Indeed, one hypothesis for APL pathogenesis suggests that PML/RARα recruits co-repressors and histone deacetylases to their putative target genes at physiological concentrations of ATRA [46, 47]. A primary effect of ATRA at pharmacological concentrations is to change the PML-RARα fusion protein from a repressor into a transcriptional activator [48]. It is therefore likely that the effect of ATRA on TF reduction is not direct but rather related to upregulation of a protein that negatively regulates TF expression. The rapid ATRA-induced reduction in TF mRNA, with a half-life of less than 1 h, is compatible with the short TF mRNA half-life previously described for TNF-stimulated endothelial cells [49]. A plausible mechanism for the effect of ATRA would therefore be an increase in TF mRNA turnover rather than a mechanism dependent on PML-RARα degradation; the latter may be relevant after ATO treatment. The rapid turnover of TF mRNA may involve an AU-rich element (ARE) in the TF mRNA molecule, which is known to recruit RNA-degrading enzymes [50]. Indeed, ARE interacting proteins such as tristetraprolin and PARP14 exert selective posttranscriptional control of TF in macrophages [51, 52].

We complemented our analysis on TF mRNA with assays of procoagulant activity of NB4 cells and of NB4 cell-derived microparticles. Several lines of evidence point to a role of microparticles emitted by leukemic cells in the disturbances of hemostasis [13, 14, 53]. These microparticles may exert a direct or an indirect procoagulant effect. Indeed, Fang et al. [54] have shown that vascular endothelial cells may uptake NB4-derived microparticles and microparticle-derived TF may make the endothelial cells procoagulant. Coagulation studies were not only the chromogenic factor Xa generation assay but also the CAT assay [35, 55]. The latter is thought to be more relevant to the in vivo phenomena, because it takes into account all factors that contribute to the pro- and anticoagulant activities of NB4 cells and microparticles [56]. The effect of ATRA on TF protein and activity is delayed with respect to its rapid effect on TF mRNA and is only evident at 24 h. Even then, significant procoagulant activity is still detected and this information is quite relevant in the context of reducing hemorrhagic complication in the early treatment phase [57]. In agreement with previous reports [58, 59], we found with the CAT assay that cells and microparticles bring both TF and an appropriate surface, since a substantial inhibition was obtained in presence of anti-TF antibody and since we did not add extra phospholipids. Of note, inhibition of thrombin generation as assessed with the CAT assay appeared to be less than that measured with the artificial Xa generation assay. This underscores the importance to assay TF activity under more physiological conditions.

Exposure of NB4 cells to ATRA resulted in a reduction of TNF mRNA, as well as an increase in IL-1β mRNA. There are previous reports on the absence of an effect of ATRA on TNF mRNA [24, 25]. It remains to be established whether the difference in effect of ATRA is due to differences in timing of TNF mRNA measurements (up to 24 h versus 2 to 4 days) or in ATRA concentration (0.5 versus 10 μM). On the other hand, the ATRA-induced increase in IL-1β expression is in agreement with previous reports [24, 25]. The effect of ATRA in reducing TNF mRNA appears to be specific for NB4 cells, because ATRA treatment of HL60 acute myeloid leukemia cells had no effect on TNF mRNA (this study) or increased TNF mRNA [24]. TNF is known to increase TF expression in different cell types. The ATRA-induced reduction of TNF mRNA occurs later than that of TF mRNA (several hours versus 30 min). Therefore, the decrease in TNF expression cannot explain the effect of ATRA on TF mRNA.

However, TNF does contribute, at least partially, to TF expression in NB4 cells because inhibition of TNF by using adalimumab consistently reduced in TF mRNA by 25% and reduced the procoagulant activity of NB4 cells and NB4 cell-derived microparticles. The contribution of IL-1β to TF mRNA was less than that of TNF, and we could not detect an effect of IL-1β inhibition on CAT activity expressed by NB4 cells and NB4 cell-derived microparticles. In APL patients, the contribution of TNF or of IL-1β may be higher because NB4 cell densities used in our experiments are much lower than the densities of leukemic cells encountered in patient’s blood and bone marrow. We were unable to study the contribution of TNF or of IL-1β at higher cell concentrations because, in our in vitro cell culture system, these are limited to approximately 10^6^ NB4 cells/ml due to acidification and exhaustion of the cell culture medium within 24 h. Leukemia cell-derived cytokines may also be relevant for thrombo-hemorrhagic events in APL patients, because they increase TF expression by endothelial cells [60–62] and monocytes [63].

We observed that inhibition of the signaling intermediates p38 or of NF-κB that act downstream of TNF or IL-1β had a more pronounced effect (38% and 62% inhibition of TF mRNA, respectively), than selective inhibition of TNF or IL-1β alone (28% and 20% inhibition of TF mRNA, respectively), whereas inhibition of jun kinase had no effect. However, it has to be stressed that inhibition of p38 results in a strong increase in TNF expression (sixfold) and an even more pronounced increase in IL-1β expression (26-fold). Thus, it appears that key elements of the signaling pathways of inflammatory cytokines are also involved in the regulation of their production. An unexpected finding of our study was that inhibition of NF-κB induced cell death within a few hours. The resulting liberation of TF-bearing microparticles may explain the increase in procoagulant activity of NB4 cell-derived microparticles, as measured with the CAT assay, despite an initial reduction in TF mRNA.

The effect of NF-κB inhibition should stimulate more detailed studies on the importance of NF-κB for APL cell survival, the mechanisms by which NF-κB inhibition induces cell death and whether NF-κB inhibition can be used as adjunct therapy in APL [64, 65]. The consequences of potential effects of NF-κB inhibition on APL cell survival should be carefully evaluated, as the apoptotic cells and the cell-derived microparticles express phosphatidylserine at their surface and may thereby exacerbate thrombo-hemorrhagic complications, despite an initial reduction in TF mRNA, but not yet in TF protein.

In conclusion, ATRA and ATO reduce expression by NB4 APL cells of both TF and TNF and inhibition of TNF or IL-1β or of the inflammatory signaling intermediates p38 or NF-κB also reduces TF.

Thus, TNF and IL-1β have a regulatory function on TF expression by NB4 APL cells, but the effect of ATRA and ATO on TF can only partially be accounted for their impact on these cytokines. The results presented here should stimulate further in vitro and in vivo preclinical studies to determine to what extent inhibition of inflammatory cytokines or inflammatory signaling intermediates can be used for the further development of adjunct therapies of APL.

## Electronic supplementary material

Supplementary Fig. 1 Time course of the effect of NF-κB inhibition on mRNA levels of TF, TNF, or IL-1β in NB4 cells. NB4 cells were exposed for different time periods to 100 μM of the NF-κB inhibitor BAY11-7085 and mRNA levels of TF, TNF, or IL-1β quantified by qPCR and expressed relative to that of the corresponding mRNA in control (unexposed cells, incubated in medium alone) NB4 cells. Results are expressed as means ± SEM of six independent experiments. (JPEG 77 kb)

High resolution image (EPS 100 kb)
