# Supplementary figures and images for: Effect of ATRA and ATO on the expression of tissue factor in NB4 acute promyelocytic leukemia cells and regulatory function of the inflammatory cytokines TNF and IL-1β
Source: Ann Hematol. 2017 Mar 25;96(6):905–17. doi: 10.1007/s00277-017-2970-5 (PMC5406437; doi:10.1007/s00277-017-2970-5)

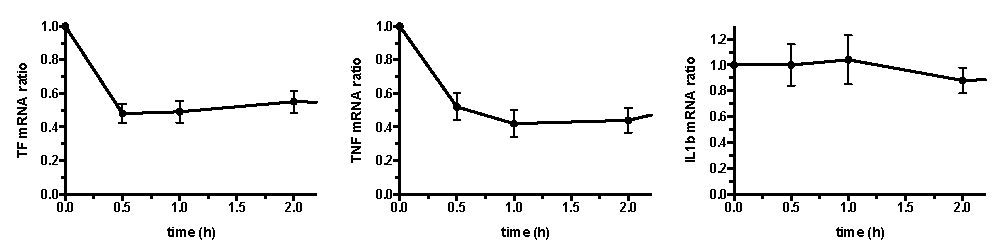

Supplement: Supplementary file 1 — Time course of the effect of NF-κB inhibition on mRNA levels of TF, TNF, or IL-1β in NB4 cells. NB4 cells were exposed for different time periods to 100 μM of the NF-κB inhibitor BAY11-7085 and mRNA levels of TF, TNF, or IL-1β quantified by qPCR and expressed relative to that of the corresponding mRNA in control (unexposed cells, incubated in medium alone) NB4 cells. Results are expressed as means ± SEM of six independent experiments. (JPEG 77 kb) [file 277_2017_2970_Fig7_ESM.jpg]
